# Drivers of rabies virus spillover risk from vampire bats to livestock in Colombia

**DOI:** 10.1371/journal.pntd.0013508

**Published:** 2025-09-26

**Authors:** Paige Van de Vuurst, Cassidy Rist, Tatiana Medina-Rodriguez, Andres Felipe Osejo-Varona, Diego Soler-Tovar, Luis E. Escobar

**Affiliations:** 1 Department of Geography and Environment, The George Washington University, Washington, District of Columbia, United States of America; 2 Translational Biology, Medicine, and Health Program, Virginia Tech Graduate School, Blacksburg, Virginia, United States of America; 3 Department of Fish and Wildlife Conservation, Virginia Tech, Blacksburg, Virginia, United States of America; 4 Center for Emerging Zoonotic and Arthropod-borne Pathogens, Virginia Tech, Blacksburg, Virginia, United States of America; 5 Department of Population Health Sciences, Virginia Tech, Blacksburg, Virginia, United States of America; 6 Technical Directorate of Epidemiological Surveillance, Deputy Directorate of Animal Protection, Colombian Agricultural Institute ICA, Bogotá, Colombia; 7 Technical Directorate of Animal Health, Deputy Directorate of Animal Protection, Colombian Agricultural Institute ICA, Bogotá, Colombia; 8 Faculty of Agricultural Sciences, Universidad de La Salle, Bogotá, Colombia; 9 Global Change Center, Virginia Tech, Blacksburg, Virginia, United States of America; 10 Kellogg Center for Philosophy, Politics, and Economics, Virginia Tech, Blacksburg, Virginia, United States of America; US Department of Agriculture, UNITED STATES OF AMERICA

## Abstract

**Background:**

Rabies is an acute and progressive viral zoonotic disease of the nervous system, which widely affects domestic animals in Latin America. Vampire bat-borne rabies virus (RABV) has significant negative impacts on the livestock industry via animal mortality. Nevertheless, the landscape level factors that facilitate or limit RABV transmission from vampire bats to livestock remain elusive.

**Methods:**

To determine how abiotic and biotic factors modulate RABV spillover from vampire bats to livestock, we assessed the role of different landscape variables on the occurrence of RABV spillover from *Desmodus rotundus* to livestock in Colombia. Using ecological niche modeling as the theoretical and analytical framework, we analyzed ecological and epidemiological RABV data to reconstruct spillover transmission events.

**Results:**

Anthropogenic variables including livestock and human density were consistently selected as predictors of RABV spillover from vampire bats to livestock. Cattle density had the highest average relative contribution to final ecological niche models (64.7%). We also found improvement of RABV spillover risk estimates when sampling bias in the form of cattle density was used in the modeling process. High risk for RABV spillover (0.75-0.98) was consistently predicted in the Caribbean region of Colombia. Nevertheless, more widespread moderate RABV spillover risk was predicted more broadly across the country when sampling bias was accounted for.

**Conclusion:**

Our modelling effort revealed that variable selection and use of bias surface have tractable impacts on final projections of spillover risk. Our results also indicate that human activity drives RABV spillover risk to a greater extent than ecological or climatological factors. Results from this study provide important information about landscape conditions linked to RABV transmission risk, where livestock vaccination should be prioritized.

## Author summary

The common vampire bat (*Desmodus rotundus*) regularly transmits rabies virus (RABV) to livestock in Latin America. Vampire-bat borne RABV causes economic losses in the livestock industry and is a threat to public health. The factors contributing to RABV spillover from vampire bats to livestock are still poorly understood, blocking progress for disease prevention. Identifying the drivers of RABV spillover could help inform prevention and control plans. To determine the factors that modulate RABV spillover, we assessed the role of different predictor variables on RABV livestock outbreaks in Colombia. We found that anthropogenic variables explained the distribution of RABV spillover more than environmental variables. Human activity expressed as habitat destruction may be driving the distribution of RABV in livestock.

## Introduction

Rabies is an acute and progressive disease of the central nervous system caused by the rabies virus (RABV) (Rhabdoviridae, genus *Lyssavirus*). Rabies is one of the oldest recorded infectious diseases to affect humans in history [1]. Despite over a century of eradication efforts and effective pre- and post-exposure vaccination, rabies has been classified as a neglected disease in several countries [2–4]. When untreated, the fatality rate of rabies is almost 100% [1,5]. There are still ~60,000 recorded human deaths due to rabies annually, mainly in Asia and Africa in areas where vaccination is limited [1,5]. In the Americas, where sanguivorous (i.e., blood feeding) bats are a key rabies reservoir [1,5,6], rabies has precipitous impacts to non-human mammals such as livestock [6–8]. In Latin America, the common vampire bat (*Desmodus rotundus)* [9] is considered to be the main wildlife species responsible for transmitting RABV to other species, including domesticated mammals [6,10–12].

Vampire bat-borne RABV outbreaks in humans and livestock regularly occur in tropical and subtropical regions of Latin America were *D. rotundus* and livestock co-occur [13], with clinical cases in humans normally following outbreaks in livestock [6]. Thousands of cattle are lost to RABV in Latin America annually, with an average of 450 outbreaks per year across the last 50 years being reported [4,14,15]. The average number of deaths per outbreak is six, but this number has been shown to vary drastically across different regions [15]. Rabies disease has been reported to cause at least US$8.6 billion in economic losses in impacted areas due to lost income and productivity worldwide [16]. Furthermore, recent years have even seen an increase in RABV livestock cases in Latin American countries [6,12,15,17], in concurrence with a range expansion of *D. rotundus* into novel areas in northern Mexico [18–20]. As such, there is a pressing need to understand factors at the local level that explain RABV spillover from vampire bats to livestock.

It has been hypothesized that abiotic factors, such as temperature, may limit the distribution of *D. rotundus*, and thus shape the geographic distribution of associated RABV spillover [21–24]. Alternately, biotic factors such as changes in vegetation primary productivity or increases in prey density may have increased the incidence of RABV spillover instead [24–26]. Nevertheless, a combination of both abiotic and biotic factors at the landscape level, such as geomorphology and landscape type, have also been shown to shape *D. rotundus* roosting and feeding behavior [27,28]. The fact that many abiotic and biotic factors have been shown to impact both *D. rotundus* and RABV spillover is indicative of the interconnected and complex nature of the factors that drive the distribution of this disease system. Due to RABV’s prolific nature, many previous modeling efforts have been conducted to reconstruct the distribution of both *D. rotundus* and RABV spillover [21–23,29,30]. Nevertheless, many previous studies have been biased toward small study areas [28] or have used few variables or only abiotic (climate) variables to assess potential distributional drivers [20,22,24]. As such, there is still a need for a comprehensive, country-level analysis of factors that shape vampire bat-borne RABV spillover to livestock at the landscape level [31].

To address this need, we conducted an ecological study to reconstruct the distribution of RABV spillover locations in Colombia using a wide variety of both abiotic and biotic environmental variables. We then assessed the relative importance of different variable types, as well as the epidemiological implications of the potential drivers as they pertain to RABV spillover risk distribution in Colombia. We hypothesized that a combination of environmental variables created using climatological, landscape and anthropological data would best predict RABV spillover risk distribution, owing to the intersectional nature of both host ecology for *D. rotundus* and the drivers for the distribution of susceptible livestock in the region. Colombia is a diverse country located at the apex of South America which houses a wide variety of ecosystems and biodiversity [32]. Colombia is also home to an expansive agricultural industry [33,34]. Recent reports from the Instituto Colombiano Agropecuario (ICA) have identified 638,941 farms across the country with over 29 million cattle [34]. While rabies pre- and post-exposure vaccination is available for both humans and animals in Colombia, RABV spillover incidence in livestock still persists, with 25 outbreaks being reported in 2023, mainly in cattle [35–37]. We therefore chose Colombia as the study area due to its expansive livestock industry and robust surveillance system for RABV in livestock.

## Methods

Geographic locations of RABV spillover to livestock data were provided by ICA [34] via the Epidemiological Information and Surveillance System in Colombia from 2014 to 2019 [37]. This organization performs surveillance for RABV livestock across Colombia via reports of suspected cases from local-level stakeholders, including farmers, independent organizations, and veterinary professionals. Suspected cases are submitted to one of the 172 local reporting offices in Colombia [29,38], and are confirmed via a laboratory direct fluorescent antibody tests [39]. RABV spillover data provided by this surveillance system included locations of farms with confirmed rabies deaths in livestock (e.g., cattle, pigs, goats, or horses), the year of outbreak, and annual number of outbreaks. For these data, an ‘outbreak’ was defined as a unique location where at least one livestock individual was confirmed to have died from RABV per year. We used the locations of farms with confirmed spillover from this dataset (n = 896) as occurrence data for our modeling effort. To account for temporal differences (i.e., lack of temporal overlap) between the collection of the environmental data and the RABV spillover locations, these occurrence points were pooled across the entire collection period (2014–2019), prior to geographic and environmental filtering.

To mitigate potential spatial sampling bias, we resampled the locations of RABV spillover occurrences to one per pixel of the study extent (11.72° N, 3.80° S, 77.37° W, 67.67° S) [40]. To mitigate overrepresentation of certain environments we also filtered our occurrence points environmentally (i.e., in environmental space) [41]. This allowed us to identify environmental outliers, or occurrence points that occurred in locations that are not usual for this system and could therefor bias our predictions. For environmental filtering we used data from all uncorrelated predictor variables to build a multidimensional environmental background where models were calibrated and where outliers could be identified [41]. Values of each predictor variable were first extracted for RABV spillover locations to create a cloud of data points representing the entire distribution of RABV spillover events in environmental space [41]. We then developed a principal component analysis (PCA) of the predictor variables to minimize dimensionality of the data, and to conceptualize a three-dimensional threshold through which outliers could be identified. The PCA allowed us to obtain three principal component axes which summarized 53.2% of the variance of the data. Within the three-dimensional environmental space created by PCA axes, we used an ellipsoid calculated using Mahalanobis distance and a precision factor of one to identify environmental outliers (i.e., those points which fell outside of the ellipsoid) from the cloud of extracted values. These points were removed from consideration during model calibration. The remaining 541 filtered occurrences were randomly split into 70% training 30% testing subsets from the thinned dataset for each model calibration and evaluation experiment.

We then compared different predictor variables to identify the combinations which provided the best model performance. Predictor variables were both grouped and ungrouped based on their characteristics (i.e., climate, landscape, or anthropogenic) (Table 1). We selected a wide array of predictor variables important to both reservoir host ecology, as well as variables suspected to be related to RABV spillover [27,36,42–44]. These included anthropogenic variables such as human population density, nighttime light, human poverty index data, and agricultural influences in the form of cattle, chicken, goat, buffalo, horse, duck, and pig density, combined-livestock density, and changes to livestock density across the last 50 years [45]. We also assessed 19 different climate variables derived from remotely sensed temperature and precipitation data [46], and an array of landscape factors including reservoir host (i.e., *D. rotundus*) density, vegetation phenology (i.e., Enhanced Vegetation Index (EVI)), continuous land cover data (Top of Atmosphere (TOA) spectral variance MERIS data), and elevation [47,48]. Anthropogenic variables were collected from the NASA Socieconomic Data and Applications Center (SEDAC) [49,50], and the Gridded Livestock of the World Database [45]. Climatic variables were collected from the WorldClim bioclimatic database [46]. Landscape variables were collected from the WorldGrids Archived database [47]. Historic *D. rotundus* density was calculated using a kernel density analysis of *D. rotundus* occurrence records from Colombia in ArcGIS Pro software (Version 2.5) [51]. Historical *D. rotundus* records data were first collected from the *Desmodus rotundus* Occurrence Record Database [52], and then filtered to include only those occurrence points which fell within continental Colombia based on their associated locational metadata. Points that fell within Colombia were then used as input points to create a continuous raster of historic *D. rotundus* distribution via the kernel density geoprocessing tool [53], which we then used as a proxy map of historic *D. rotundus* density.

**Table 1:** Variable type summary. List and experiment names of each background environmental variable used in each modeling experiment. Predictor variables were grouped based on their abiotic, biotic, and anthropogenic characteristics. Variables with Pearson Correlation coefficient greater than 0.5 were not grouped together in their respective experimental subsets, and removed from the “All Uncorrelated Variables” group.

Predictor Variable Sets	Variable Names	Units	Original Spatial Resolution	Sources
Abiotic Climate Variables	Isothermality (Bio3) Minimum Temperature of the Coldest Month (Bio6) Annual Precipitation (Bio12) Precipitation Seasonality (Bio15)	*°C* *°C* *mm* *mm*	*30 Arc Seconds* *30 Arc Seconds* *30 Arc Seconds* *30 Arc Seconds*	*WorldClim Bioclimatic Variables of the World Dataset* [46]
Biotic Anthropogenic Variables	Cattle Density Chicken Density Human Population Density Poverty Index	*Individuals per km* ^*2*^ *Individuals per km* ^*2*^ *Index value (1–100)*	*5 Arc Minutes* *5 Arc Minutes* *1 km* ^*2*^	*Gridded Livestock of the World database* [45] *WorldGrids Archived Database* [47] *Gridded Relative Deprivation Index* [50]
Abiotic and Biotic Landscape Variables	Elevation Enhanced Vegetation Index (EVI) Standard Deviation Continuous Land Cover *Desmodus rotundus* density	*Meters* *Index value (0–1)* *TOA Reflectance* *Individuals per km* ^*2*^	*30 Arc Seconds* *1 km* ^*2*^ *30 Arc Seconds* *1 km* ^*2*^	*WorldGrids Archived Database* [47] Derived from *Desmodus rotundus Occurrence Record Database* [52]
All Uncorrelated Variables	Minimum Temperature of the Coldest Month (Bio6) Annual Precipitation (Bio12) Enhanced Vegetation Index (EVI) Standard Deviation Continuous Land Cover *Desmodus rotundus* density Poverty Index	*°C* *mm* *Index Value (0–1)* *TOA Reflectance* *Individuals per km* ^*2*^ *Index value (1–100)*	*30 Arc Seconds* *30 Arc Seconds* *1 km* ^*2*^ *30 Arc Seconds* *1 km* ^*2*^ *1 km* ^*2*^	*WorldClim Bioclimatic Variables of the World Dataset* [46] *WorldGrids Archived Database* [47] Derived from *Desmodus rotundus Occurrence Record Database* [52] *Gridded Relative Deprivation Index* [50]

All predictor variables were collected or resampled to 1 km resolution with the World Geodetic System 1984 (WGS84) reference system and cropped to a rectangular study area that encompassed continental Colombia (11.72° N, 3.80° S, 77.37° W, 67.67° S) in R (Version 4.1.0). Pixels within the study area that fell within the Pacific Ocean or Caribbean Sea were classified as “no data”, and therefore were removed from consideration during the model calibration process. Predictor variables from each characteristic group (i.e., climate, landscape, and anthropogenic) were compared using a Pearson Correlation coefficient analysis to identify variables which were correlated [41,54]. Variables with a correlation coefficient greater than 0.5 were classified as highly correlated, as the closer the correlation coefficient gets to 1, the greater the linear relationship between the two variables is [41,54,55]. Inclusion of colinear variables has been shown to hinder transferability in MaxEnt models [55], so highly correlated variables were eliminated from predictor variable sets based on their importance to the vampire bat-livestock RABV system [44–46]. Variable selection was based on the assumption that eliminating correlated predictor variables would reduce overfitting in predictions and mitigate collinearity effects [56]. Variables that were removed from consideration included nighttime light, goat, buffalo, horse, duck, and pig density, combined-livestock density, changes to livestock density across the last 50 years, and 15 of the 19 climate variables. We then compared all remaining predictor variables regardless of characteristic, and removed correlated variables from the “All Uncorrelated Variables” experimental group (Table 1). We created suites of all possible combinations of each predictor variable set (Table 1), by removing and replacing each variable (i.e., jackknifing) and then removing redundant combinations. The best combination of variables for each predictor experiment was selected during the calibration and evaluation process.

Model calibration, evaluation, and projection was done using MaxEnt [57] version 3.4.4 in R statistical software version 4.1.0 using the *kuenm* package [58]. MaxEnt is a presence-background comparison-based ecological niche modelling algorithm frequently used in scientific literature [59–61], which does not require true absence locations. MaxEnt has built in parameterizations (e.g., regularization multiplier and feature classes), which can be altered and evaluated to identify the best potential combination of these parameters in terms of the resultant model’s predictive capacity [62]. The regularization multiplier defines how precisely the output distribution is fitted, penalizing model over-fit and is set to one as a default within the software [63]. Feature classes within MaxEnt are used as a mathematical transformation of the original predictor variables, which allows for more complex relationship to be identified [63]. To elucidate the impact of regularization multipliers and feature classes within MaxEnt on the outcome of each predictor variable experiment, we tested a suite of regularization multipliers above and below default (i.e., 0.01, 0.1, 0.5, 1, 2, 5, and 10), and all possible combinations of the available continuous feature classes (i.e., linear, product, quadratic, threshold, and hinge). The *kuenm* package allows for systematic comparison between different predictor variables sets, regularization multipliers, and feature classes, and thus allows for a systematic and holistic comparison between different iterations of the desired model. Furthermore, MaxEnt also allows for the incorporation of bias surfaces, which manipulate the background sampling effort within the algorithm via a density raster which represents relative sampling effort, thus accounting for possible sampling bias [64]. We tested the use of cattle density [45] and road accessibility [47] surfaces as possible drivers of sampling bias, as we assumed both of these factors potentially shape the surveillance of RABV spillover in Colombia. Parameters for each experiment were tested in terms of model fit and prediction as follows (Table 2).

**Table 2:** Final models summary. Summary of significant models (those which fit Omission Rate and AICc criteria) and final models selected by MaxEnt. Final models selected by MaxEnt are identified by the algorithm using pROC, omission rate, and AICc. RM denotes regularization multipliers of final selected models. FC denotes feature classes of final selected models.

Predictor Variable Set Experiments	Significant Candidate Models (p < 0.05, E < 0.05)	N Final Models Selected by MaxEnt	Final Model(s) Parameters	Final Model Predictor Variables	Fractional Predicted Area (minimum training presence)	Fractional Predicted Area (10^th^ percentile training presence)
All Variables, Ungrouped	3167	1	RM-1 FC- lqp	All variables but Poverty Index	0.95	0.35
Climate Only	680	1	RM-5 FC-pth	Isothermality (Bio3) Minimum Temperature of the Coldest Month (Bio6) Annual Precipitation (Bio12) Precipitation Seasonality (Bio15)	0.99	0.66
Anthropogenic Only	649	2	RM-2 FC-lt,lqt	Cattle Density Chicken Density Human Population Density Poverty Index	0.98	0.47
Landscape Only	632	1	RM-1 FC-lpt	Elevation Enhanced Vegetation Index Standard Deviation Continuous Land Cover	0.98	0.62
Grouped Variables	172	2	RM-2 FC-lt,lqt	Cattle Density Chicken Density Human Population Density Poverty Index	0.98	0.47
Grouped, Corrected with Accessibility	284	2	RM-5 FC-t,pt	Cattle Density Chicken Density Human Population Density Poverty Index	0.94	0.35
Grouped, Corrected with Cattle Density	87	2	RM-5 FC-lt, lpt	Chicken Density Human Population Density Poverty Index	0.98	0.59

After creating all possible interactions of candidate models for each experiment, we used the function *kuenm_ceval*, which evaluates model performance based on statistical significance using partial ROC (pROC), omission rate (*E* = 0.05) [65], and model complexity Akaike values (AICc) [58]. pROC and omission rates were calculated based on models created with training data only, whereas AICc values are calculated for models created with training and testing data [59]. pROC values were utilized to isolate statistically significant models, which also met the omission rate criteria (*E* = 0.05, 500 iterations, *p* < 0.05) [58]. These models resembling robust predictive performance were used to compare across predictor variable combinations with an analysis of variance and post hoc Fisher’s least significance difference (LSD) test. We also recorded and reported the regularization multiplier and feature classes most frequently present in statically significant models which met the omission rate criteria. We used *kuenm_eval* to select best models based on our user-set criteria with the lowest AICc, which were then used to project each final model from each experiment to the geographic extent of our study area. Model parameterizations (i.e., variable combination, regularization multipliers, and features classes) with more than one best model were averaged using the logistic continuous model outputs from MaxEnt. Logistic continuous model outputs from MaxEnt were also used to delineate and quantify suitability or similarity of model outputs to areas of known spillover risk (i.e., locations of known spillover used as occurrence data for model calibration) [62]. The “MaxEnt suitability index”, which ranges from zero to one, can therefore be interpreted as suitability for RABV spillover risk or environmental similarity to areas of known previous RABV spillover outbreaks. In other words, the higher the value of MaxEnt suitability index, the higher the RABV spillover risk of that given area. High risk was quantified as any value above 0.75, moderate risk was classified as any value between 0.25 and 0.50, and low risk was classified as any value below 0.25. These thresholds were based on previous assessments of bovine rabies distribution in Colombia, and based on institutional standards from ICA as they pertain to RABV spillover risk projections [36,38]. We then recorded the total area of the study extent reported as “suitable” (fractional predicted area) of each best model using two threshold values: (i) minimum training presences threshold and (ii) a 10^th^ percentile training presence threshold [41] as the absolute lowest value of suitability for RABV spillover risk.

## Results

**Fig 1:**
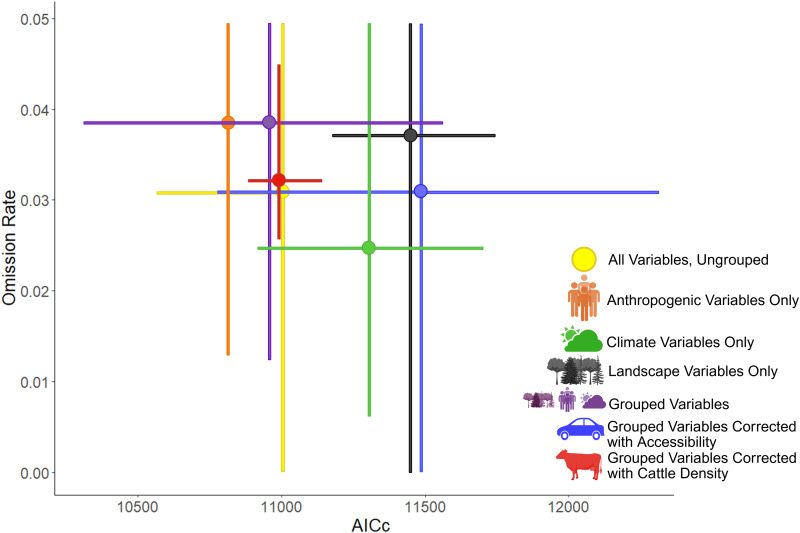
Model experiment performance. Model performance in terms of omission rate and AICc for each set of predictor variable experiments. Predictor variables for each experiment are shown as: all variables (yellow), climate only (green), landscape only (black), anthropogenic only (orange), grouped variables (purple), and grouped variables with bias surface correction (red and blue). Models calibrated using only climate variables had the overall lowest omission rates (green). Models calibrated with anthropogenic variables which used cattle density as a bias file; however, had the lowest amount of variance in both omission rates and AICc (red). This figure was created in BioRender. Van de Vuurst, P. (2025) https://BioRender.com/0b3muw.

**Fig 2:**
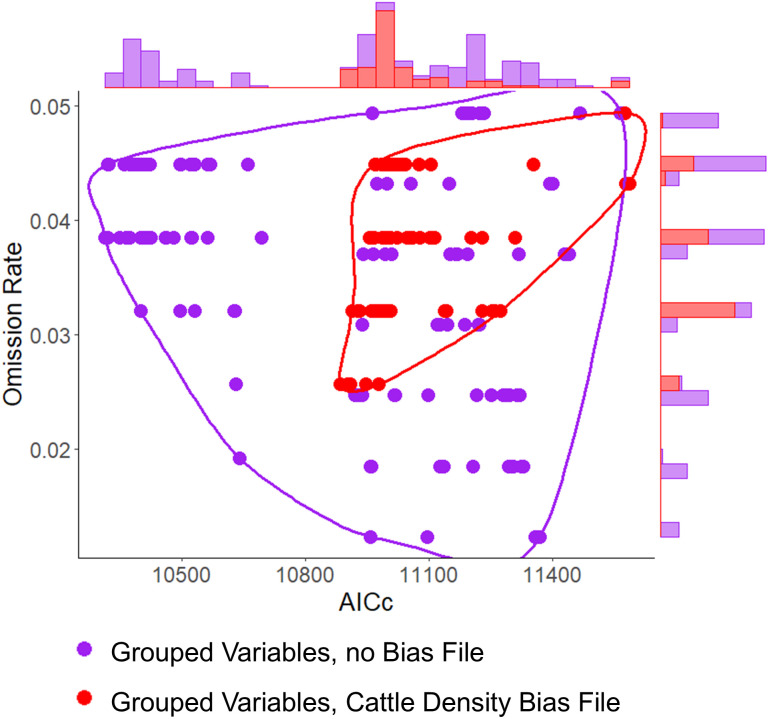
Bias file impact on model performance. The overall reduction in model performance uncertainty between models without sampling bias correction (purple), and models which accounted for sampling bias (red) highlights the importance of accounting for sampling bias in ecological niche modeling. While models without bias correction were more well preforming (i.e., low omission rate and AICc of Purple points), model performance was more precise when bias was taken into account (red points). Sampling bias was corrected using cattle density data from Gridded Livestock of the World database [45]. Models which accounted for sampling bias using cattle density (red) had a lower amount of variance in both omission rates (sd = 0.006 vs 0.0110) and AICc (sd = 139 vs 395), with the differences in AICc being statistically significant per post hoc LSD analysis (*p* < 0.001).

Each RABV model parameterization generated statistically significant predictions of independent localities with spillover reports (Table 2). Models calibrated using only climate variables had the lowest errors in terms of omission rates, and models calibrated using anthropogenic variables only had the lowest complexity in terms of AICc values (Fig 1). Models calibrated with grouped variables and cattle density as a bias surface had higher stability in the form of the lowest amount of variance in both omission rates and AICc (Fig 2). AICc was significantly different in models with cattle density as a bias surface per a post hoc LSD analysis (std = 139.4, df = 5664, r = 87, *p* < 0.001). Using road accessibility as a bias surface did not improve model performance (Fig 1).

**Fig 3:**
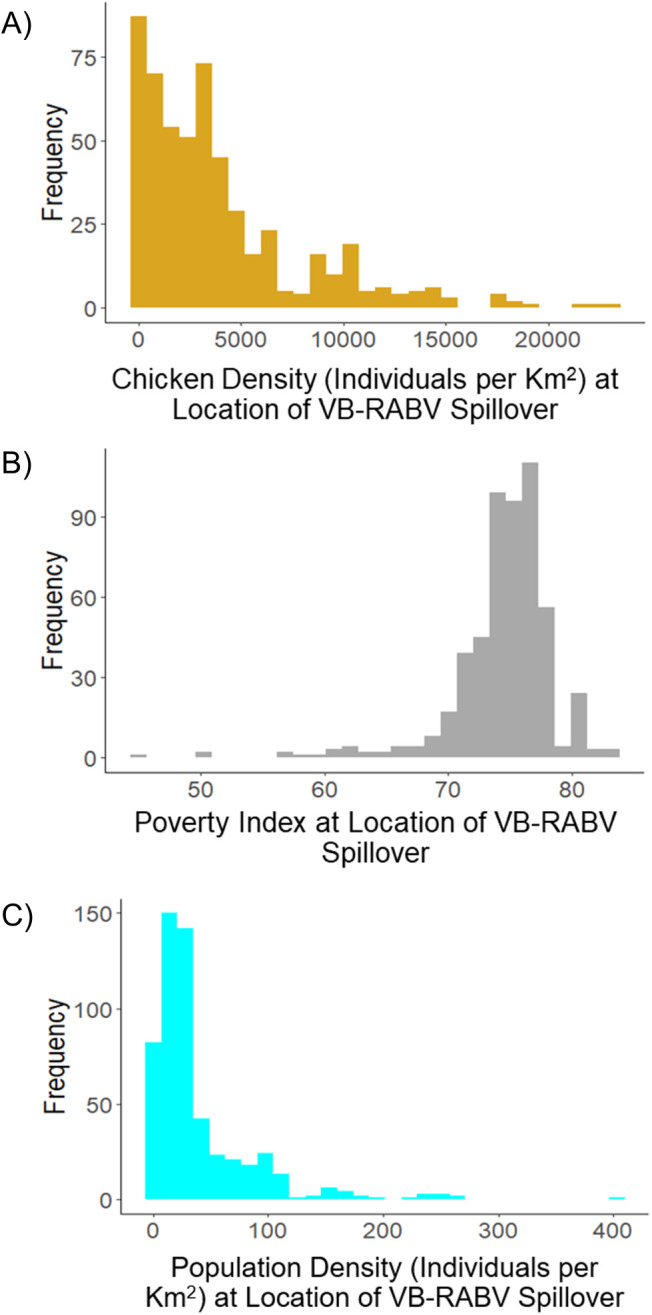
Anthropogenic predictor variable relationships to RABV. A) Histogram of chicken density (number of individual chickens per square kilometer) at locations of RABV spillover used as training and testing data. Note the negative relationship between chicken density and RABV spillover locations. B) Histogram of poverty index at locations of RABV spillover used as training locations. Note the positive association between poverty index and RABV spillover locations. C) Histogram of human population density (number of individuals per km^2^) at locations of RABV spillover used as training locations. Note the negative association between human population density and RABV locations. These results indicate that RABV spillover risk is higher when chicken density is low, poverty index is high, and human population density is low. The majority of RABV spillover occurred in locations with fewer than 5000 chickens per km^2^, human density was less than 100 per km^2^, and where poverty index was greater than 70.

Anthropogenic variables such as livestock density, human population density, and poverty index provided the best predictive performance (Table 2). Cattle density had the highest average relative contribution (i.e., percent contribution to final MaxEnt model) (64.7%) when the variable was not used as bias surface. Of the remaining anthropogenic variables, chicken density had the highest relative contribution (39.98%) to RABV spillover distribution, followed by human population density (10.55%), and poverty index (1.27%). In contrast to poverty index and cattle density, there was a negative association between chicken density and human population density and the continuous log suitability for RABV suitability (Fig 3). The bulk of RABV spillover risk occurred when chicken density was less than 5000 animals per km^2^, and where poverty index was greater than 70 (Fig 3).

For climate-only models, precipitation seasonality had the highest percent contribution (44.5%) followed by annual precipitation (39.4%), minimum temperature of the coldest month (8.4%), and isothermality (7.7%). Using only landscape variables resulted in continuous land cover data having the highest percent contribution (40.5%), followed by Enhanced Vegetation Index (EVI) standard deviation (34.3%), and elevation (25.2%). The only variable not present in any final model was historic *D. rotundus* density. For experiments with predictor variables grouped by their characteristics (i.e., climate, landscape, or anthropogenic), anthropogenic variables were consistently selected as the best set of predictor variables with and without sample bias correction. Climate-only models and models using cattle density as a bias surface had the smallest difference in fractional predicted area (i.e., area predicted to be at risk) between minimum training presence and 10^th^ percentile training presence (Table 2). The lack of change in fractional predicted area indicates that climate models and models with cattle density bias file correction had the most consistent predictions regardless of the threshold used to convert models from continuous into binary. Anthropogenic variables (i.e., chicken density and human population density) consistently explained the likelihood of spillover to a greater extent than climate or landscape variables alone.

**Fig 4:**
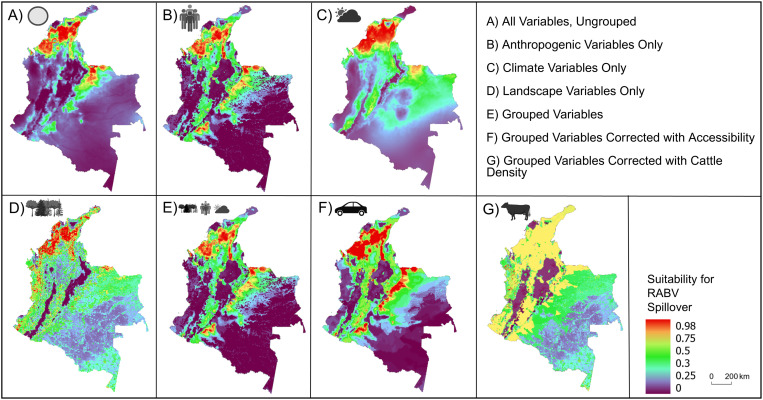
Geographic projection of each final model per predictor variable model experiment. Similarity of projected pixel to presence locations (i.e., suitability for RABV spillover) is shown from low (purple) to high (red). Maps correspond to predictor variable groups used including A) all uncorrelated variables, B) anthropogenic variables only, C) climate variables only, D) landscape variables only, E) all variables grouped by characteristics, F) all variables grouped by characteristic and with accessibility as a bias surface, and G) all variables grouped by characteristic and with cattle density as a bias surface. Predictor variable sets are broken down in Table 2. Note the differences between model outputs when cattle density data are used as a predictor variable vs as a sampling bias correction file (G). The total area predicted to have moderately or moderately-high suitability (yellow) was much larger (30.6% of the total area) when sampling bias was accounted for in panel G. High suitability for RABV spillover (red) was localized to northern portions of the country when only climate variables were used (C) than when anthropogenic (B) or landscape (D) variables were used. Maps created using ArcGIS Pro software version 2.5 with shape files from DIVA-GIS [51,84]. This figure was created using BioRender. Van de vuurst, P. https://BioRender.com/0b3muw1.

Spatial clusters of RABV transmission risk were predicted in the northern most portions of Colombia and lowland areas to the east of the Andes Mountain range (Fig 4). These areas fall within the Caribbean, Andes, and Orinoquía regions of Colombia, where higher numbers of RABV spillover outbreaks have been reported previously [29,66]. High RABV spillover risk (0.75-0.98 MaxEnt suitability index) was consistently predicted in the departments of Cordoba, Sucre, Bolivar, Magdalena, Cesar, and Arauca (Fig 4). There was low predicted RABV spillover risk (0.25-0 MaxEnt suitability index) in the Amazon and Pacific regions of Colombia (Fig 4). Though the patterns of projected suitability for RABV spillover were consistent between models, the magnitude of spillover suitability risk in certain areas differed (Fig 4). The total area predicted to have moderately or moderately-high suitability (0.5-0.75 MaxEnt suitability index) was larger (30.6% of the total area) in the model that used cattle density as a bias surface (Fig 4).

## Discussion

To identify the intersectional drivers of pathogen spillover from vampire bats to livestock, we compared the role of different predictor variables in explaining the spatial risk of RABV outbreaks in Colombia. Anthropogenic variables more accurately explained the geographic distribution of RABV spillover than climatic and landscape variables alone. These results could indicate that spatial epidemiology models using anthropogenic variables may be useful to refine RABV transmission risk estimates. Climate-only models demonstrated the lowest omission rates of each experiment, suggesting overall predictive power at the cost of losing spatial detail by predicting large areas of risk. For a zoonotic pathogen of high lethality (100%), such as RABV, omission rates may be of more priority than commission rates, which can be interpreted as overestimation of risk instead of underestimation of it. Using climate variables only in ecological niche modeling is a common practice, though recent literature has highlighted the importance of including biotic variables for more accurate prediction of biodiversity distributions [43]. Our results indicate that the use of only abiotic variables, as is common in spatial epidemiology, may be inaccurate to predict zoonotic spillover risk.

Cattle density was an important variable in predicting areas with RABV spillover, as cattle comprise the majority of identified RABV cases in livestock in Colombia [34,36]. The importance of this variable echoes previous research in Colombia, which found that number of cattle correlated with changes in RABV spillover expansion [29]. Chicken density also proved to be an informative variable, especially when cattle density was used as a bias surface rather than as a predictor variable. The likelihood of chicken density directly driving RABV spillover risk is however, unlikely due to its limited relationship to the epidemiology of RABV [67]. In fact, there was a negative association between suitability for RABV spillover and chicken density (Fig 3). That is, risk of RABV spillover to livestock was greater in areas where chickens were scarce. Chickens are not considered to be a preferred prey species for *D. rotundus* and are not susceptible to RABV, but vampire bats have been known to occasionally feed on chickens [67,68]. In fact, another recent study identified that *D. rotundus* blood meals were biased toward chickens when assessed in an urban environment, and bat-human attacks were aggregated in locations where chicken blood meals were identified from stomach contents [69]. It may be possible that chickens are acting as an alternative prey source for sanguivorous bats when they are present in high densities, thus diluting the impact of RABV spillover on cattle nearby.

Alternatively, chicken density could be functioning as a proxy of socioeconomic conditions in rural Colombia in our results. More specifically, chicken density could fluctuate in association with other factors such landscape perturbation, human presence, or subsistence agriculture [44] which could be impacting RABV spillover risk in Colombia. Low chicken density has been linked to poverty, food insecurity, and income disparity, in contrast with more developed areas with industrial poultry farming [70,71]. Rural poverty, food insecurity, and income disparity have all previously been linked to RABV spillover risk as well [72]. Here, low-density (i.e., scattered chickens) could be a proxy of subsistence farming and, in turn, poverty, which could explain why high RABV risk was found in areas with low chicken density and high poverty (Fig 3). The relationship between high RABV spillover risk and the anthropogenic factors identified by this study in Colombia likely reflects the interconnected nature of socioeconomic and environmental factors that are present in small villages, such as those in rural settlements, more likely to experience *D. rotundus* depredation. Untangling the specifics within the anthropogenic factors that may drive RABV spillover distribution such as poverty, livestock practices, and the spatiotemporal distribution of these factors would be a beneficial next step for this system.

We also identified differences in the magnitude of RABV spillover risk projection when sampling bias was included in the models. We assumed that cattle industry was a driver of RABV surveillance in Colombia and thus considered the density of cattle as a source of bias. Estimates of RABV spillover risk created using cattle density as bias surface identified larger proportional areas of risk, with lower redundancy with areas of known spillover (Fig 4). Mitigating sampling bias in this way revealed that areas of Colombia at risk for RABV spillover were more generalized (Fig 4). As such, RABV spillover risk may be more widespread than previously identified. For example, lowland areas in central portions of the country were identified as having equal RABV spillover risk to more northern areas of the country where cattle density is higher. Another recent paper assessing RABV spillover risk found that there was no relationship between RABV prevalence and increased cattle density as was previously hypothesized [73], which supports our findings. Furthermore, previous research also found a negative association between livestock density and RABV seroprevalence in vampire bats within the context of landscape change [74]. Increases in deforestation, rather than cattle density, was also identified as a driving factor in increased bovine RABV outbreaks in Costa Rica [44], and previous RABV epidemics in Trinidad were associated with land use patterns rather than livestock density [75]. These results, coupled with our assessment, could indicate that generalized landscape disruption associated with anthropogenic activity is a greater predictor of RABV spillover risk than ecological factors associated with vampire bats alone. Nevertheless, a shift in the distribution of cattle density or associated landscape changes could influence a spatial shift in the incidence and distribution of RABV spillover risk in Colombia, especially if surveillance remains focused on high cattle density areas. Overall, our study demonstrates that the decision of using a variable as a predictor or as a sampling bias surface has a strong effect on the final risk estimates produced. Modelers should consider the biological meaning of the data when deciding if a variable can be used as a predictor or as a proxy of bias.

While our assessment highlights specific points that may guide a further elucidation of the intersectional factors that shape RABV spillover risk distribution, this study did have some notable limitations. The spatial risk of RABV spillover identified here is temporally static and does not account for bat dispersal potential or waves of disease spread [76]. A year-to-year assessment of RABV spillover in Colombia and vampire bat behavior, in conjunction with annual level environmental variables, may allow for a finer identification of driving factors which this study was not able to do. Furthermore, it is possible that disparities in data quality or reporting consistency may have impacted the distribution of RABV spillover data used as occurrence points for this assessment. Nevertheless, the spatial and environmental filtering of the spillover locations prior to model calibration, as well as the use of multiple bias files likely mitigated this potential impact. Fine-scale studies of *D. rotundus* movement are also still needed to better quantify how bat-population connectivity and dispersal may affect RABV spillover dynamics [77]. It has been hypothesized that colony or roost level factors, such as seasonal reproduction or dispersal, may influence RABV spillover frequency [18,78–80]. These factors were not accounted for in this study and should be investigated in Colombia to further our understanding of this disease system. Finally, our assessment relied upon surveillance of rabies-associated livestock deaths, which could have underestimated the frequency of spillover events in Colombia. Recent research has identified that abortive RABV infectious in *D. rotundus* may be more common, leading to an even more complex disease spread system than previously identified [76,81]. An exploration of RABV seroprevalence in *D. rotundus* individuals themselves, in conjuncture with mortality in livestock, may capture a broader picture of spillover dynamics in this region. Future research should consider investigating seroprevalence of *D. rotundus* individuals in Colombia across gradients of livestock density or environmental conditions, which may help refine the patterns of spillover risk identified in this study.

In Colombia, RABV outbreaks in livestock increased dramatically between 2010 and 2019 [29,36,82,83]. The country has also seen an increase in RABV incidence outside of previously endemic areas as well in recent years [29,82], indicating that RABV may be changing in association with some of the factors we identified. Extensive vaccination efforts are needed across endemic and non-endemic regions for effective rabies prevention in Latin America [29]. As such, our results could guide vaccination efforts toward areas that were consistently identified as having high risk across all models, such as the Caribbean region. Other regions with more variability between projected RABV spillover risk, such as the Pacific or Orinoquía regions, may require different management strategies. For example, rather than broad sweeping vaccination or surveillance across the entire area, more targeted intervention may be used in locations with conditions consistent with those identified in this study as being susceptible to RABV spillover. These would include smaller farms with susceptible livestock, higher poverty, and potentially in close association with anthropogenic landscape perturbation.

## Conclusions

Results of this study suggest that anthropogenic factors, like livestock density, are informative to anticipate RABV spillover risk over environmental or climatic data. From an analytical perspective, our results highlight the importance of predictor variable selection (e.g., combination of predictor variables and control of sampling bias) in spillover risk estimations. In a more applied context, our modeling efforts show that human agricultural practices may ultimately drive RABV spillover risk, rather than environmental conditions alone. Future research on RABV spillover should further explore the impacts of anthropogenic factors on RABV transmission at the local level, such as socioeconomic conditions and human behavior. Ecological niche models created for this study revealed a wide distribution of RABV spillover risk in Colombia. This study provides important information about conditions linked to transmission risk and reveals hotspots of RABV risk where livestock vaccination could be prioritized.
